# 8-Azaadenosine and 8-Chloroadenosine are not Selective Inhibitors of ADAR

**DOI:** 10.1158/2767-9764.CRC-21-0027

**Published:** 2021-11-02

**Authors:** Kyle A. Cottrell, Luisangely Soto-Torres, Michael G. Dizon, Jason D. Weber

**Affiliations:** 1 Department of Medicine, Division of Molecular Oncology, Siteman Cancer Center, Washington University School of Medicine, Saint Louis, Missouri.; 2 Department of Cell Biology and Physiology, Siteman Cancer Center, Washington University School of Medicine, Saint Louis, Missouri.

## Abstract

The RNA editing enzyme ADAR is an attractive therapeutic target for multiple cancers. Through its deaminase activity, ADAR edits adenosine to inosine in double-stranded RNAs. Loss of ADAR in some cancer cell lines causes activation of the type I IFN pathway and the PKR translational repressor, leading to inhibition of proliferation and stimulation of cell death. As such, inhibition of ADAR function is a viable therapeutic strategy for many cancers. However, there are no FDA-approved inhibitors of ADAR. Two small molecules have been previously shown to inhibit ADAR or reduce its expression: 8-azaadenosine and 8-chloroadenosine. Here we show that neither molecule is a selective inhibitor of ADAR. Both 8-azaadenosine and 8-chloroadenosine show similar toxicity to ADAR-dependent and -independent cancer cell lines. Furthermore, the toxicity of both small molecules is comparable between cell lines with either knockdown or overexpression of ADAR, and cells with unperturbed ADAR expression. Treatment with neither molecule causes activation of PKR. Finally, treatment with either molecule has no effect on A-to-I editing of multiple ADAR substrates. Together, these data show that 8-azaadenosine and 8-chloroadenosine are not suitable small molecules for therapies that require selective inhibition of ADAR, and neither should be used in preclinical studies as ADAR inhibitors.

**Significance:**

ADAR is a good therapeutic target for multiple cancers; neither 8-chloroadenosine nor 8-azaadenosine are selective inhibitors of ADAR.

## Introduction

ADAR (encoded by *ADAR*, also known as ADAR1 or DSRAD) carries out adenosine-to-inosine (A-to-I) editing within double-stranded RNA (dsRNA; refs. 1–5). By editing dsRNA, it has been proposed that ADAR prevents sensing of self dsRNAs by dsRNA-binding proteins involved in activation of the type I IFN response and/or control of translation (6–10). Depletion of ADAR in numerous cancer cell lines causes reduced proliferation and increased apoptosis (11–14). Consistent with its proposed role in preventing dsRNA sensing, loss of ADAR in many human cancer cell lines leads to activation of the type I IFN pathway through activation of MAVS and translation repression by activation of PKR (11–13). The growth phenotype of ADAR depletion can be rescued by disruption of type I IFN signaling or knockdown of PKR (11–13). Because of the importance of ADAR expression in many human cancer cell lines, several groups have proposed the use of ADAR inhibitors as a therapy for lung, breast, and thyroid cancers (11–14).

**FIGURE 1:**
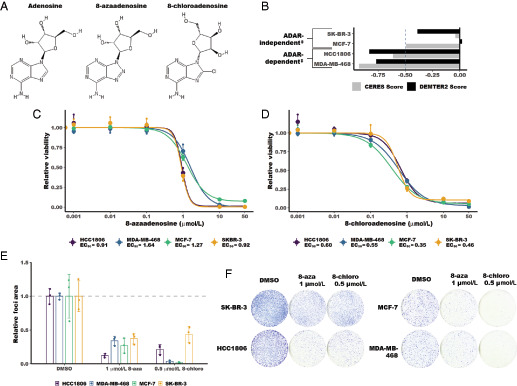
8-chloroadenosine and 8-azaadenosine inhibit proliferation of ADAR-dependent and ADAR-independent breast cancer cell lines. **A,** Structure of adenosine, 8-azaadenosine, and 8-chloroadenosine. **B,** A graph summarizing the ADAR dependency status of relevant breast cancer cell lines as previously published. DEMETER2 corresponds to ADAR dependency as determined by RNAi screening (21–23). CERES corresponds to ADAR-dependency as determined by CRISPR-Cas9 screening (24, 25). A DEMETER2 or CERES score of less than −0.5 is considered “dependent” or “essential” (21, 24). The ^‡^ symbol indicates ADAR-dependency status as determined previously (13). **C,** Dose–response curve for 8-azaadenosine treatment of several breast cancer cell lines. **D,** Dose–response curve for 8-chloroadenosine treatment of several breast cancer cell lines. In **C** and **D,** cell viability was measured by CellTiter-Glo 2.0. **E,** Quantification of foci formation. **F,** Following treatment of several breast cancer cell lines with 8-chloroadenosine (8-chloro) or 8-azaadenosine (8-aza). For all panels, error bars are mean ± SD. In **C** and **D,** the large points are the mean of three independent experiments, and the smaller points are the mean of three technical replicates performed for each experiment. For **E,** the smaller points represent the relative foci area from each of three independent experiments and the column represents the mean foci area of the three experiments.

There are currently no FDA-approved ADAR inhibitors. However, two small molecules have previously been reported to either inhibit ADAR or reduce its expression (14–16). Both of these small molecules are adenosine analogues (Fig. 1A). 8-azaadenosine has been used as an ADAR inhibitor in multiple studies involving leukemic stem cells and thyroid cancer cell lines (14, 16). In thyroid cancer cell lines, 8-azaadenosine has been shown to be very effective at inhibiting proliferation, even at doses as low as 1–2 μmol/L (14). The use of 8-azaadenosine as an inhibitor of ADAR was initially inspired by a study that incorporated 8-azaadenosine and other adenosine analogues into an ADAR substrate to identify modified substrates that would serve to resolve the structure of ADAR (17). In that study, it was observed that an ADAR substrate containing 8-azaadenosine resulted in improved A-to-I editing (17). As such, it is conceivable that free 8-azaadenosine could serve as a competitive inhibitor of ADAR.

Another adenosine analogue, 8-chloroadenosine, has been shown not to inhibit the deaminase activity of ADAR itself, but to reduce ADAR expression (15). Treatment of several breast cancer cell lines with 8-chloroadenosine led to reduced ADAR expression and induction of cell death. The cell death phenotype could be rescued by overexpression of wild-type ADAR, but not a dsRNA binding–deficient mutant of ADAR, suggesting that 8-chloroadenosine could have some selectivity toward ADAR.

Here we set out to further evaluate the therapeutic potential of 8-chloroadenosine and 8-azaadenosine as ADAR inhibitors. Using several approaches, we show that neither 8-chloroadenosine nor 8-azaadenosine are selective inhibitors of ADAR: both molecules inhibited growth of ADAR-depleted cells, treatment with neither molecule caused activation of PKR, and treatment with neither molecule reduced A-to-I editing of multiple ADAR substrates. Together, these results do not support the use of 8-azaadenosine or 8-chloroadenosine as ADAR inhibitors, and instead warrant the future search for novel ADAR inhibitors.

## Materials and Methods

### Cell Culture

Breast cancer cell lines [MCF-7 (RRID:CVCL_0031), SK-BR-3 (RRID:CVCL_0033), HCC1806 (RRID:CVCL_1258), MDA-MB-468 (RRID:CVCL_0419)] were obtained from ATCC in 2011. Cell lines have not been authenticated since purchase from the manufacturer. *Mycoplasma* testing was performed PCR in October of 2019 prior to freeze-back. Cell lines were passaged for at least one month prior to the experiments described here. All cell lines were cultured in DMEM (Hyclone) with 10% FBS (Invitrogen), 2 mmol/L glutamine (Hyclone), 0.1 mmol/L nonessential amino acids (Hyclone), 1 mmol/L sodium pyruvate (Hyclone), and 2 μg/mL gentamicin (Invitrogen). 8-chloroadenosine and 8-azaadenosine were purchased from Tocis (catalog numbers: 4436 and 6868).

### Viral Production and Transduction

Lentivirus was produced by Turbo DNAfectin 3000 (Lambda Biotech) transfection of 293T cells with pCMV-VSV-G, pCMV-ΔR8.2, and pLKO.1-puro for short hairpin RNA (shRNA). Virus was harvested 48 hours posttransfection. Cells were transduced with lentivirus for 16 hours in the presence of 10 μg/mL protamine sulfate. The cells were selected with puromycin at 2 μg/mL for one day. For analysis of ADAR expression and PKR activation following ADAR knockdown, cells were harvested 96 hours after transduction. The sequences for the shRNA-scramble (shSCR) and shADAR were described and validated previously (13). For ADAR overexpression cell lines, SK-BR-3 cells were transduced with lentivirus produced from pLVX-IRES-Hygro-EV, pLVX-IRES-Hygro-p110 or pLVX-IRES-Hygro-p150 described previously (18).

### Immunoblot

Cell pellets were lysed and sonicated in RIPA Buffer (50 mmol/L Tris pH 7.4, 150 mmol/L NaCl, 1% Triton X-100, 0.1% SDS and 0.5% sodium deoxycholate) with 1× HALT Protease Inhibitor (Pierce). Forty micrograms of protein lysate were resolved on 4%–12% TGX Acrylamide Stain-Free gels (Bio-Rad). Proteins were transferred to polyvinylidene difluoride membrane (Millipore). The membrane was cut into strips corresponding to the molecular weight of proteins of interest. The blots were blocked and then probed with the appropriate primary antibodies: ADAR1 (Santa Cruz Biotechnology, sc-73408), PKR (Cell Signaling Technology, #3072), PKR Thr-446-P (Abcam, ab32036), GAPDH (Bethyl Laboratories, A300–641A). Primary antibodies were detected with horseradish peroxidase–conjugated secondary antibodies (Jackson ImmunoResearch) and detection was carried out with Clarity Western ECL Substrate (Bio-Rad). Chemiluminescence was imaged using a ChemiDoc imaging system (Bio-Rad). Quantification of immunoblots was performed using Image Lab software (Bio-Rad). The abundance of each protein was normalized to GAPDH abundance. For PKR and pPKR, two separate gels were resolved, transferred, and probed for either PKR or pPKR in addition to GAPDH for both. PKR and pPKR abundance were normalized to GAPDH prior to normalizing pPKR to PKR. Uncropped immunoblot images are available in Supplementary Figs. S1–S5.

### Analysis of A-to-I Editing

Cells were treated as indicated for 72 hours prior to harvesting of RNA using the Nucleospin RNA kit (Macherey-Nagel). First-strand cDNA synthesis was performed using iScript Supermix (Bio-Rad). The cDNA was purified using the Monarch DNA and PCR Cleanup Kit (New England Biolabs). Regions around A-to-I editing sites in BPNT1, MRPS16, or ZDHHC20 were amplified by Q5 Hot Start High-Fidelity 2X Master Mix (New England Biolabs) and the primers: BPNT1_F 5′-TGCTGTGGGAGGCAAGTTAAC-3′ and BPNT1_R 5′-GAGTCCGAGGCAGACAGATC-3′, or MRPS16_F 5′-GAAATCGCACACTGAAATATCC-3′ and MRPS16_R 5′-TTGACTCACAACCATTCTTAGGTC-3′, or ZDHHC20_F 5′-TGCTGTACTAGGAAATGACAGAGC-3′ and ZDHHC20_R 5′-AACATTCTGTGATGCCTAATTTTG-3′. For PCR of the BPNT1 editing site, the parameters were as follows: 98°C for 30 seconds, 98°C for 30 seconds, 72°C for 30 seconds, 72°C for 55 seconds, repeat steps 2–4 for 19 cycles dropping the annealing temperature 0.2°C each cycle, 98°C for 30 seconds, 68°C for 30 seconds, 72°C for 55 seconds, repeat steps 6–8 for 19 cycles, 72°C for 5 minutes. For PCR of the MRPS16 and ZDHHC20 editing sites, the parameters were as follows: 98°C for 30 seconds, 98°C for 30 seconds, 60°C for 30 seconds, 72°C for 30 seconds, repeat steps 2–4 for 39 cycles, 72°C for 5 minutes. The PCR products were resolved by agarose gel electrophoresis and purified using the Monarch Gel Extraction kit (New England Biolabs). Purified PCR products were Sanger sequenced by Genewiz using either the BPNT1_F_Seq primer: 5′-GGAGTCTCGCTCTGTAGCCT-3′, MRPS16_F or ZDHHC20_F. The chromatograms for all replicates are available in Supplementary Figs. S6–S14. To determine percent editing, raw peak heights were measured for the edited and unedited base using the program QSVanalyzer (19). Percent editing was calculated by the following formula for BPNT1:



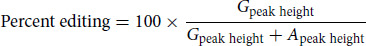



For MRPS16 and ZDHHC20, the primer used for sequencing resulted in a sequence that is the reverse complement of the mRNA sequence, thus editing was determined by the ratio of T (unedited) to C (edited). Percent editing was calculated by the following formula for MRPS16 and ZDHHC20:



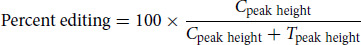



#### Quantitative PCR

First-strand cDNA synthesis was performed using iScript Supermix (Bio-Rad) with RNA isolated for analysis of A-to-I editing (described above). For qPCR, the primers listed below were used with iTaq SYBR Green (Bio-Rad). Fold change in RNA expression was determined by the ΔΔ*C*_t_ method with normalization to GAPDH and HPRT1. Primers for qPCR: GAPDH_F 5′-GAGTCAACGGATTTGGTCGT-3′, GAPDH_R 5′-GACAAGCTTCCCGTTCTCAG-3′, HPRT1_F 5′-CTTCCTCCTCCTGAGCAGTC-3′, HPRT1_R 5′-TCACTAATCACGACGCCAGG-3′, CMPK2_F 5′-GGCCAACAGTGTGTTTCGTC-3′, CMPK2_R 5′-CTTTTCTCTGGAGGGGCTGG-3′, CXCL10_F 5′-GAACCTCCAGTCTCAGCACC-3′, CXCL10_F 5′-GATGCAGGTACAGCGTACAG-3′.

### Measurement of Cell Viability

Cells were treated as indicated for 96 hours prior to assessment of cell viability using CellTiter-Glo 2.0 (Promega) per manufacturers’ protocol. Luminescence was measured for 10 seconds using a Promega Glomax Navigator luminometer. Dose–response analysis was performed using the R package "drc" (20). A four-parameter log-logistic model (LL.4) was fit to the viability data. For this log-logistic model, the Hill Coefficient, lower limit, and EC_50_ were allowed to vary but the upper limit was set to 1. Further details for this analysis can be found in the GitHub repository below.

### Foci Formation Assay

Five thousand cells were plated for each condition in a 10-cm culture dish. Three days later, the cells were treated as indicated. After 9 (HCC1806 and SK-BR-3) to 14 (MCF-7 and MDA-MB-468) days, the cells were washed briefly with 1× PBS prior to fixation in 100% methanol. After drying, the cells were stained with Giemmsa (Sigma-Aldrich) prior to washing excess stain away with deionized water. The plates were scanned using an ImageScanner III (General Electric). Foci area was calculated using ImageJ.

### Data Availability Statement

Scripts used for all plots are available on GitHub (https://github.com/cottrellka/ADAR_5–2021). The data generated in this study are available within the article and its Supplementary Data files. Data from DepMap used in Fig. 1B can be obtained here: https://depmap.org/portal/download/.

## Results

### Cytotoxicity of 8-Chloroadenosine and 8-Azaadenosine in Breast Cancer Cell Lines

Knockdown or knockout of ADAR causes reduced proliferation and increased cell death in numerous, but not all cancer cell lines (11–14). ADAR dependency has been evaluated through large screening experiments (21–25) and smaller studies involving knockdown or knockout of ADAR in panels of human cancer cell lines (11–14). Recently, ADAR dependency was evaluated for a panel of human breast cancer cell lines (13). To evaluate the on-target effects of 8-chloroadenosine and 8-azaadenosine, we assessed the effects of each small molecule on cell viability of breast cancer cell lines previously identified to be ADAR-dependent or -independent (Fig. 1B). If 8-chloroadenosine and/or 8-azaadenosine are selective inhibitors of ADAR, it would be expected that the EC_50_ for cell viability of each drug would be lower for ADAR-dependent cell lines relative to ADAR-independent cell lines. However, analysis of the effects of each adenosine analogue on cell viability found that the EC_50_s were comparable between ADAR-dependent and independent cell lines, Fig. 1C–D. For 8-chloroadenosine there was an approximately 0.25 μmol/L EC_50_ difference between the most sensitive cell line (MCF-7, ADAR-independent) and the least (HCC1806, ADAR-dependent). Similarly, for 8-azaadenosine there was a < 1 μmol/L EC_50_ difference between the most sensitive cell line (SK-BR-3, ADAR-independent) and least sensitive (MDA-MB-468, ADAR-dependent). These data were largely supported by foci formation analysis (Fig. 1E–F). The ADAR-independent cell lines SK-BR-3 and MCF-7, and the ADAR-dependent cell line MDA-MB-468 were similarly sensitive to the effects of 8-azaadenosine on foci formation. The two cell lines most sensitive to the effects of 8-chloroadenosine on foci formation were MCF-7 and MDA-MB-468, ADAR-independent and ADAR-dependent cell lines, respectively. Taken together, these data show that neither 8-chloroadenosine nor 8-azaadenosine are selectively cytotoxic toward ADAR-dependent cell lines.

### Cytotoxicity of 8-Chloroadenosine and 8-Azaadenosine in ADAR-Depleted or ADAR-Overexpressed Cells

**FIGURE 2:**
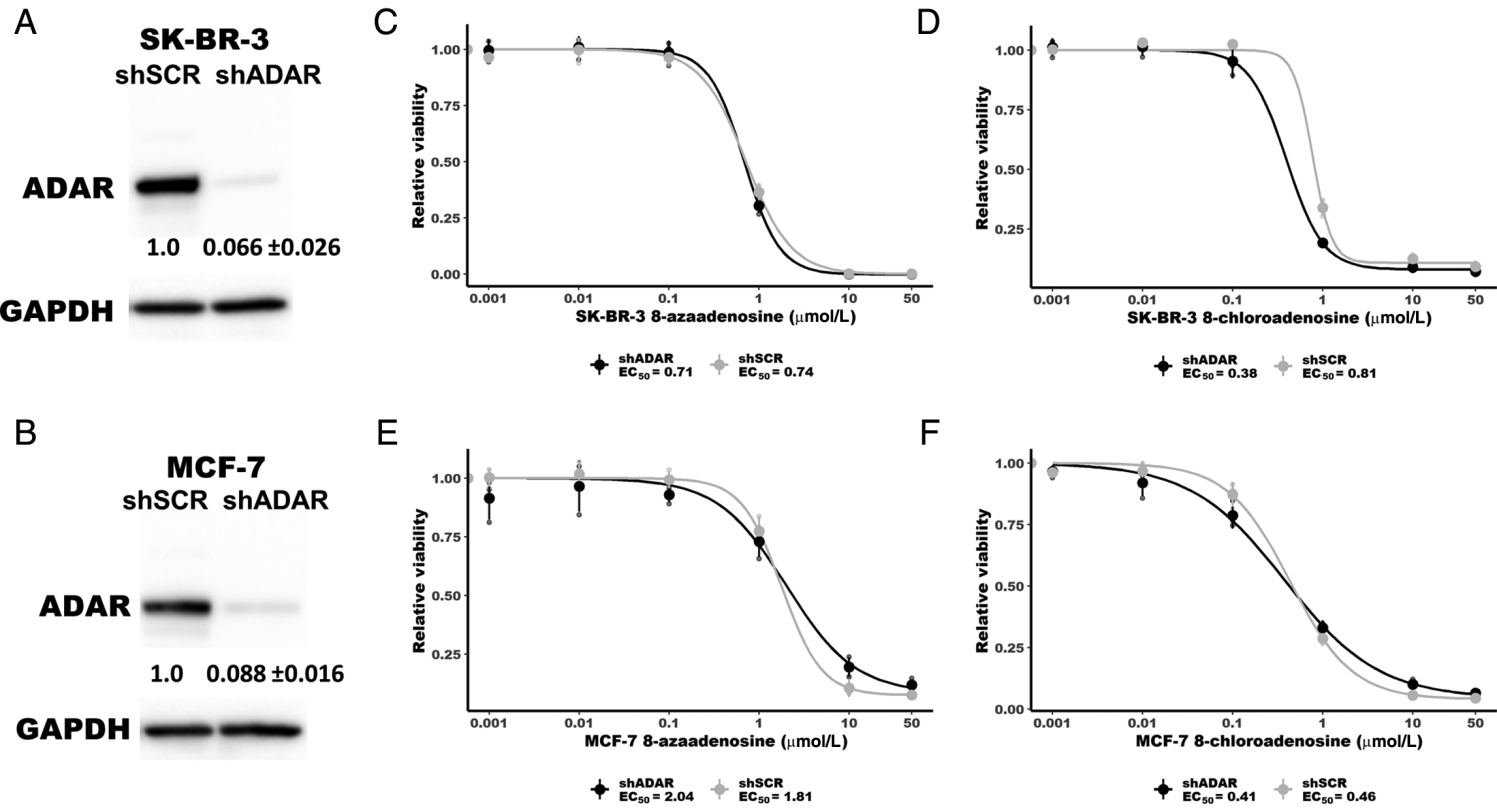
8-chloroadenosine and 8-azaadenosine inhibit proliferation of ADAR-depleted breast cancer cell lines. Immunoblot of ADAR knockdown in SK-BR-3 (**A**) and MCF-7 (**B**). The level of ADAR knockdown is shown below each band, mean ± SD. Five (SK-BR-3) or six (MCF-7) days after transduction of shSCR or shADAR, the cells were treated with 8-chloroadenosine or 8-azaadenosine for dose response curves. **C** and **D,** Dose–response curves for 8-azaadenosine and 8-chloroadenosine in SK-BR-3 cells with (shADAR) or without (shSCR) ADAR knockdown. **E** and **F,** Dose–response curves for 8-azaadenosine and 8-chloroadenosine in MCF-7 cells with (shADAR) or without (shSCR) ADAR knockdown. In **C–F,** the large points are the mean of three independent experiments, the smaller points are the mean of three technical replicates performed for each experiment, error bars are mean ± SD.

While the data described in Fig. 1 are consistent with 8-azaadenosine and 8-chloroadenosine lacking selectivity for ADAR, we sought to address this question more thoroughly by assessing the cytotoxicity of the small molecules in ADAR-depleted cell lines. ADAR was knocked-down in two ADAR-independent cell lines, SK-BR-3 and MCF-7, Fig. 2A and 2B. The EC_50_ of cell viability for 8-azaadenosine and 8-chloroadeonsine was evaluated for control (shSCR) or ADAR knockdown (shADAR). If 8-azaadenosine and/or 8-chloroadenosine are selective inhibitors of ADAR, it would be expected that ADAR-depleted cells would be less sensitive to each adenosine analogues. However, the EC_50_ for each drug was generally similar between shSCR and shADAR transduced cells for both cell lines, Fig. 2C–D and 2E–F. Only for 8-chloroadenosine was there a clear difference between the EC_50_ in shSCR versus shADAR transduced cells, with shADAR cells having a lower EC_50_.

Since ADAR expression is often elevated in cancer (13, 26, 27), we assessed the cytotoxicity of 8-chloroadenosine and 8-azaadenosine in SK-BR-3 with or without overexpression of the p110 or p150 isoforms of ADAR. The EC_50_ of 8-azaadenosine and 8-chloroadeonsine was similar between empty vector (EV), p110 and p150 overexpressed SK-BR-3, Supplementary Fig. S15. Together these data, and the data in Fig. 1, show that neither 8-chloroadenosine nor 8-azaadenosone induce cytotoxicity through selective inhibition of ADAR.

### Treatment with 8-Chloroadenosine or 8-Azaadenosine does not Activate PKR

**FIGURE 3:**
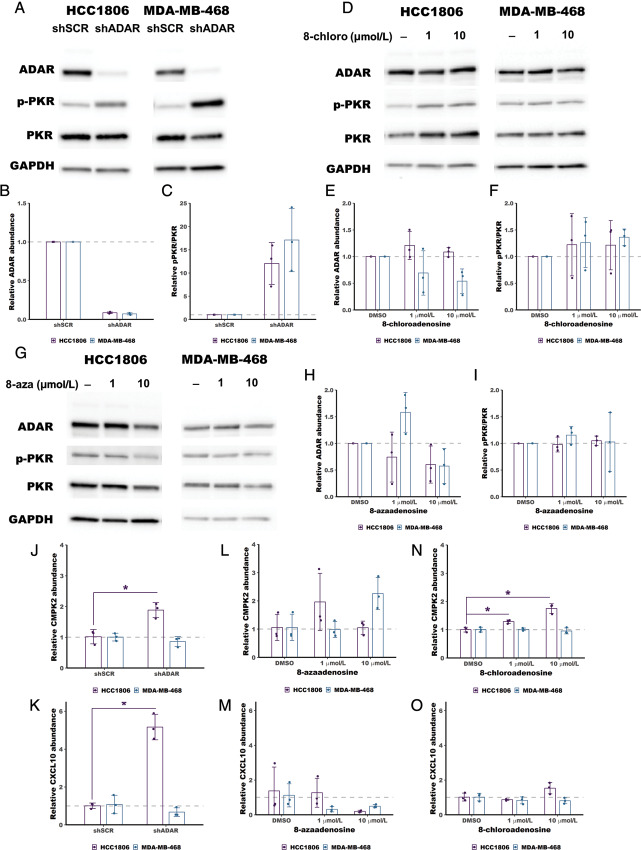
Treatment with 8-chloroadenosine or 8-azaadenosine does not activate PKR. **A,** Immunoblot showing activation of PKR (increased phosphorylation of PKR at T446, pPKR) following knockdown of ADAR in HCC1806 and MDA-MB-468. **B** and **C,** Quantification of the immunoblot in **A**. **D,** Immunoblot showing no activation of PKR following treatment of HCC1806 or MDA-MB-468 with 8-chloroadenosine (8-chloro). **E** and **F,** quantification of the immunoblot in **B**. **G,** Immunoblot showing no activation of PKR following treatment of HCC1806 or MDA-MB-468 with 8-azaadenosine (8-aza). **H** and **I,** Quantification of the immunoblot in **G**. **J** and **K,** qPCR showing increased ISG expression following knockdown of ADAR in HCC1806 but not MDA-MB-468. **L–O,** qPCR assessment of ISG expression following treatment with 8-azaadenosine or 8-chloroadenosine in HCC1806 and MDA-MB-468. For **B**, **C**, **E**, **F,** and **J–O,** the smaller points represent relative ADAR abundance, relative pPKR/PKR, relative CMPK2 abundance or relative CXCL10 abundance from each of three independent experiments, and the column represents the mean of the three experiments. Error bars are mean ± SD.*, *P* < 0.05; *t* test.

Loss of ADAR in ADAR-dependent cells has been shown to cause activation of the dsRNA sensor PKR (11–13). It has been proposed that loss of A-to-I editing by ADAR causes accumulation of dsRNA leading to activation and autophosphorylation of PKR (9). Activated PKR represses translation and can induce cell death (11–13). Selective inhibitors of ADAR would be expected to also cause significant PKR activation. We evaluated PKR activation upon treatment with 8-chloroadenosine or 8-azaadenosine by immunoblot using a phospho-PKR (phospho-T446) specific antibody. Unlike knockdown of ADAR, which caused robust activation of PKR in the ADAR-dependent cell lines HCC1806 and MDA-MB-468 (Fig. 3A–C), neither 8-chloroadenosine nor 8-azaadenosine induced PKR activation in the same cell lines, Fig. 3D–I. Furthermore, treatment with neither adenosine analogue caused increased expression of PKR (Supplementary Figs. S2 and S4). These data suggest that neither 8-chloroadenosine nor 8-azaadenosine are inhibitors of ADAR.

### Treatment with 8-Chloroadenosine or 8-Azaadenosine does not Phenocopy the Effects of ADAR Knockdown on ISG Expression

Loss of ADAR in ADAR-dependent cells has been shown to cause activation of the type I IFN pathway through activation of MDA5 due to accumulation of dsRNA (6, 10). Activation of the type I IFN pathway induces transcription of several IFN-stimulated genes (ISG). Selective inhibitors of ADAR would be expected to also cause type I IFN pathway activation and induced ISG expression. We evaluated the expression of two ISGs (CMPK2 and CXCL10) previously shown to be induced upon ADAR depletion (11). Knockdown of ADAR induced expression of both ISGs in HCC1806, while no induction was observed in MDA-MB-468 (Fig. 3J and K). Unlike knockdown of ADAR, treatment with 8-azaadenosine did not cause induction of either ISG in HCC1806 (Fig. 3L and M). Treatment with 8-chloroadenosine induced CMPK2 expression in HCC1806, consistent with ADAR knockdown in that cell line (Fig. 3N). However, unlike knockdown of ADAR, treatment with 8-chloroadenosine did not induce expression of CXCL10 (Fig. 3O). Taken together, these data suggest that neither 8-chloroadenosine nor 8-azaadenosine phenocopy the effects of ADAR knockdown on ISG expression.

### Treatment with 8-Chloroadenosine or 8-Azaadenosine has no Effect on A-to-I Editing

**FIGURE 4:**
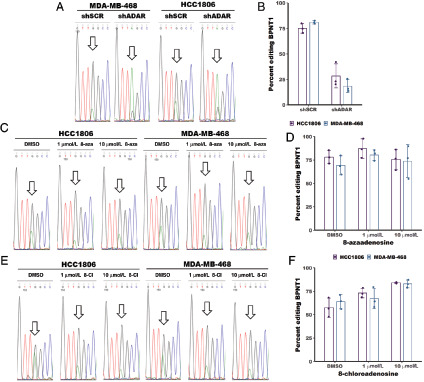

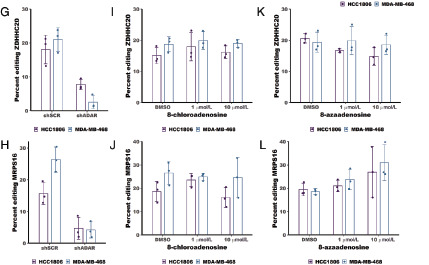
Treatment with 8-chloroadenosine or 8-azaadenosine does not affect A-to-I editing. **A,** Sanger sequencing chromatogram of BPNT1 with or without ADAR knockdown. The arrow indicates a base edited by ADAR. The editing site is at position 1894 within the BPNT1 transcript (NM_006085.6). **B,** Quantification of percent editing as measured by Sanger sequencing in **A**. Percent editing was calculated as the edited base (G) peak height divided by the total peak height of the unedited (A) and edited (G) base. **C,** Sanger sequencing chromatogram of BPNT1 with or without 8-azaadenosine (8-aza) treatment. **D,** Quantification of percent editing from **C**. **E,** Sanger sequencing chromatogram of BPNT1 with or without 8-azaadenosine (8-aza) treatment. **F,** Quantification of editing efficiency from **E**. **G**, **I**, and **K,** Quantification of percent editing for the editing site at position 2877 in the ZDHHC20 transcript (NM_153251.4) following knockdown of ADAR or treatment with 8-azaadenosine or 8-chloroadenosine in HCC1806 and MDA-MB-468. **H**, **J**, and **L,** Quantification of percent editing for the editing site at position 2231 in the MRPS16 transcript (NM_016065.4) following knockdown of ADAR or treatment with 8-azaadenosine or 8-chloroadenosine in HCC1806 and MDA-MB-468. For **B**, **D**, and **F–L,** the smaller points represent percent editing from each of three independent experiments, and the column represents the mean of the three experiments. Error bars are mean ± SD.

To directly test the effects of 8-azaadenoinse and 8-chloroadenosine on the deaminase activity of ADAR, we used Sanger sequencing to measure A-to-I editing of a highly edited ADAR substrate – BPNT1 (28). The adenosine at position 1894 in the BPNT1 mRNA was shown to be highly edited (∼75%) in four different breast cancer cell lines (28). Percent editing can be measured by Sanger sequencing of PCR amplified cDNA. As inosine pairs most readily with cytosine, reverse transcriptase will incorporate a cytosine at each A-to-I editing event. Sanger sequencing of the PCR product made from the cDNA will show either an A (for unedited transcripts) or a G (for edited transcripts). We performed this analysis to assess the change in A-to-I editing of BPNT1-A1894 upon ADAR knockdown. Knockdown of ADAR reduced editing by approximately 3-fold (Fig. 4A and B). The same analysis was performed for cells treated with either 1 or 10 μmol/L 8-azaadenosine or 8-chloroadenosine. There were no substantial changes to editing of BPNT1-A1894 upon treatment with either adenosine analogue (Fig. 4C–F). To extend these findings, we assessed editing of two additional A-to-I editing sites previously shown to be highly edited in breast cancer cell lines – MRPS16-A2231 and ZDHHC20-A2877 (28). Like the BPNT1-A1894 site, knockdown of ADAR caused reduce editing of both MRPS16-A2231 and ZDHHC20-A2877 (Fig. 4G and H). However, there were no substantial changes to editing of either site upon treatment with 1 or 10 μmol/L 8-azaadenosine or 8-chloroadenosine in either MDA-MB-468 or HCC1806 cell lines (Fig. 4I–L). Together, these data clearly show that neither 8-chloroadenosine nor 8-azaadenosine affects A-to-I editing of three separate editing sites.

## Discussion

Several recent studies have highlighted the importance of ADAR expression in a wide range of cancer cell lines (11–14). In ADAR-dependent cells, loss of ADAR causes activation of PKR and the type I IFN pathway leading to reduced proliferation and apoptosis. Furthermore, depletion of ADAR in cell lines that do not require ADAR expression to grow in tissue culture conditions has been shown to improve antitumor immunity *in vivo*, especially in combination with anti–PD-1 therapies (29). The importance of ADAR in tumor biology therefore makes it an ideal therapeutic target for multiple cancers.

While there are currently no FDA-approved ADAR inhibitors available for clinical use, two adenosine analogues have been used in preclinical studies to perturb ADAR activity or expression – 8-chloroadenosine and 8-azaadenosine (14–16). We found that both adenosine analogues efficiently reduce the viability of both ADAR-dependent and ADAR-independent cell lines. Similarly, both adenosine analogues reduced the viability of ADAR-depleted or ADAR-overexpressed cell lines to a similar or greater extent than cell lines with unperturbed ADAR expression. We showed that treatment with neither 8-chloroadenosine nor 8-azaadenosine caused activation of PKR, in contrast with ADAR knockdown which caused robust PKR activation in the same cell lines. While treatment with 8-chloroadenosine induced expression of one ISG (CMPK2), it did not phenocopy the effects of ADAR knockdown. Because the type I IFN pathway can be activated in multiple ways, it is possible that the effect observed with 8-chloroadenosine is not due to dsRNA accumulation caused by inhibition of ADAR, which would be consistent with the lack of PKR activation upon 8-chloroadenosine treatment. Finally, we observed that neither adenosine analogue inhibited A-to-I editing of multiple ADAR substrates.

The off-target effects of either 8-chloroadenosine or 8-azaadenosine are consistent with what is known about the biological activity of both adenosine analogues. It has been shown that both adenosine analogues can be incorporated into nascent RNA and DNA (30–32), and inhibit DNA synthesis (31, 33). Furthermore, both 8-azaadenosine and 8-chloroadenosine can be rapidly incorporated into the cellular ATP pool, replacing ATP with 8-azaATP or 8-chloroATP (32–35). 8-chloroadenosine has also been shown to cause inhibition of mTOR and activation of AMPK in renal cell carcinoma cell lines (36). In addition, 8-chloroadenosine has been shown to activate the unfolded protein response leading to apoptosis in coronary artery endothelial cells (32). Finally, *in vivo* studies of 8-azaadenosine toxicity revealed significant hepatic toxicity (34). Taken together, these previous findings, along with those presented here, show that 8-chloroadenosine or 8-azaadenosine likely cause cell death through numerous indirect effects and not through selective inhibition of ADAR. Neither 8-azaadenosine nor 8-chloroadenosine should be used as ADAR inhibitors.

## Supplementary Material

Supplementary Data Supplementary Data
