# Supplementary material for: 8-Azaadenosine and 8-Chloroadenosine are not Selective Inhibitors of ADAR
Source: Cancer Res Commun. 2021 Nov 2;1(2):56–64. doi: 10.1158/2767-9764.CRC-21-0027 (PMC9113518; doi:10.1158/2767-9764.CRC-21-0027)
Supplement: Supplementary Data [file crc-21-0027-s01.pdf]

Supplemental Information for:

8-azaadenosine and 8-chloroadenosine are not selective inhibitors of ADAR

Kyle A. Cottrell[1], Luisangely Soto Torres[1], Michael G. Dizon[1], Jason D. Weber[1,2,*]

[1]Department of Medicine, Division of Molecular Oncology and [2]Department of Cell Biology and Physiology, Siteman Cancer Center, Washington University School of Medicine, Saint Louis, Missouri, USA
*Corresponding author

Correspondence:
Jason D. Weber, Ph.D.
Department of Medicine
Division of Molecular Oncology
Washington University School of Medicine
660 South Euclid Avenue
Campus Box 8069
St. Louis, MO 63110 USA
Email: jweber@wustl.edu

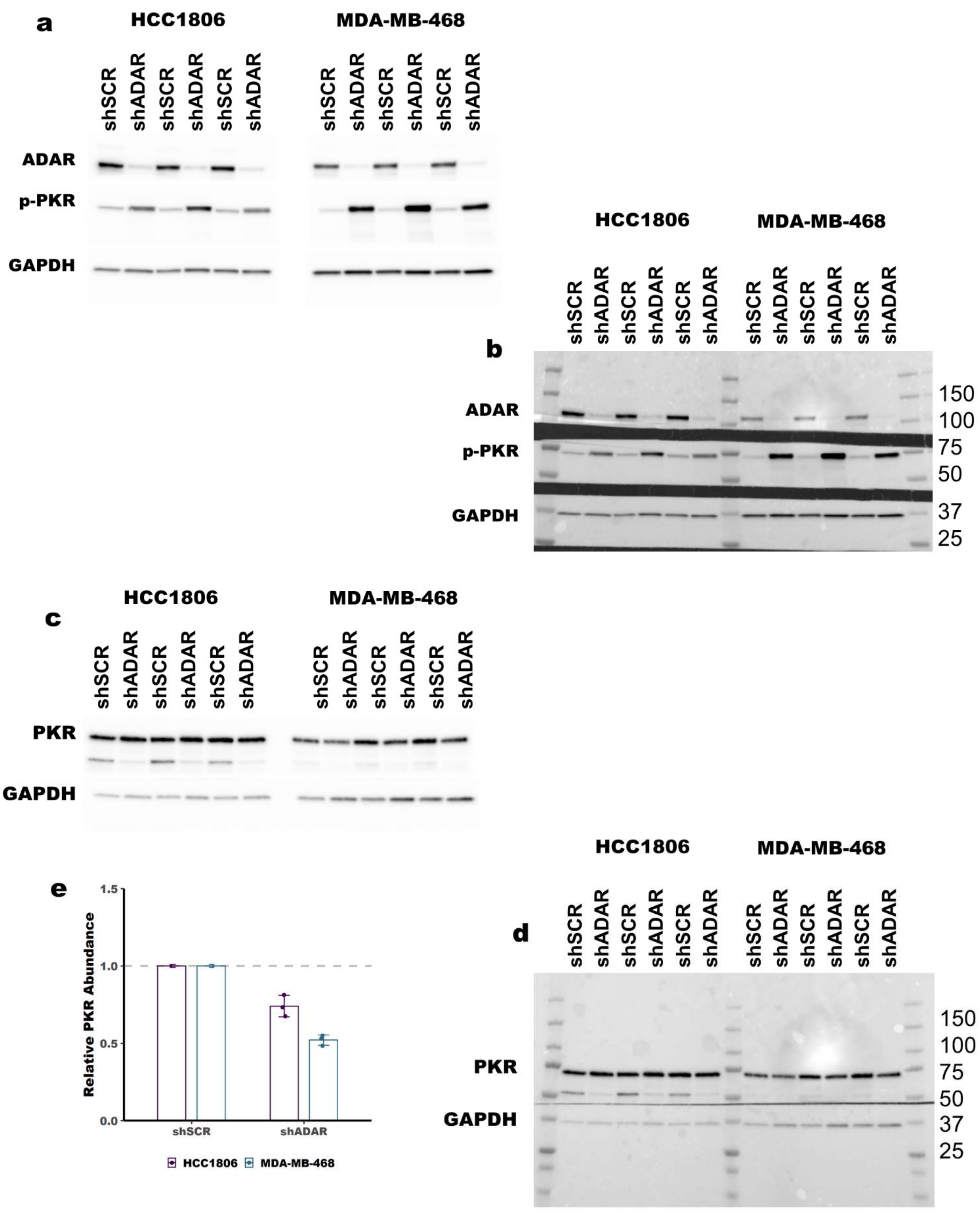

Supplemental Figure 1:

Uncropped immunoblots associated with Figure 3A, 3C and 3D. Panels **a** and **b** are the uncropped chemiluminescence images, panels **b** and **d** are the chemiluminescence images merged with colorimetric images to show the molecular weight marker. **e** Quantification of PKR expression, PKR abundance was normalized to GAPDH and set relative shSCR.

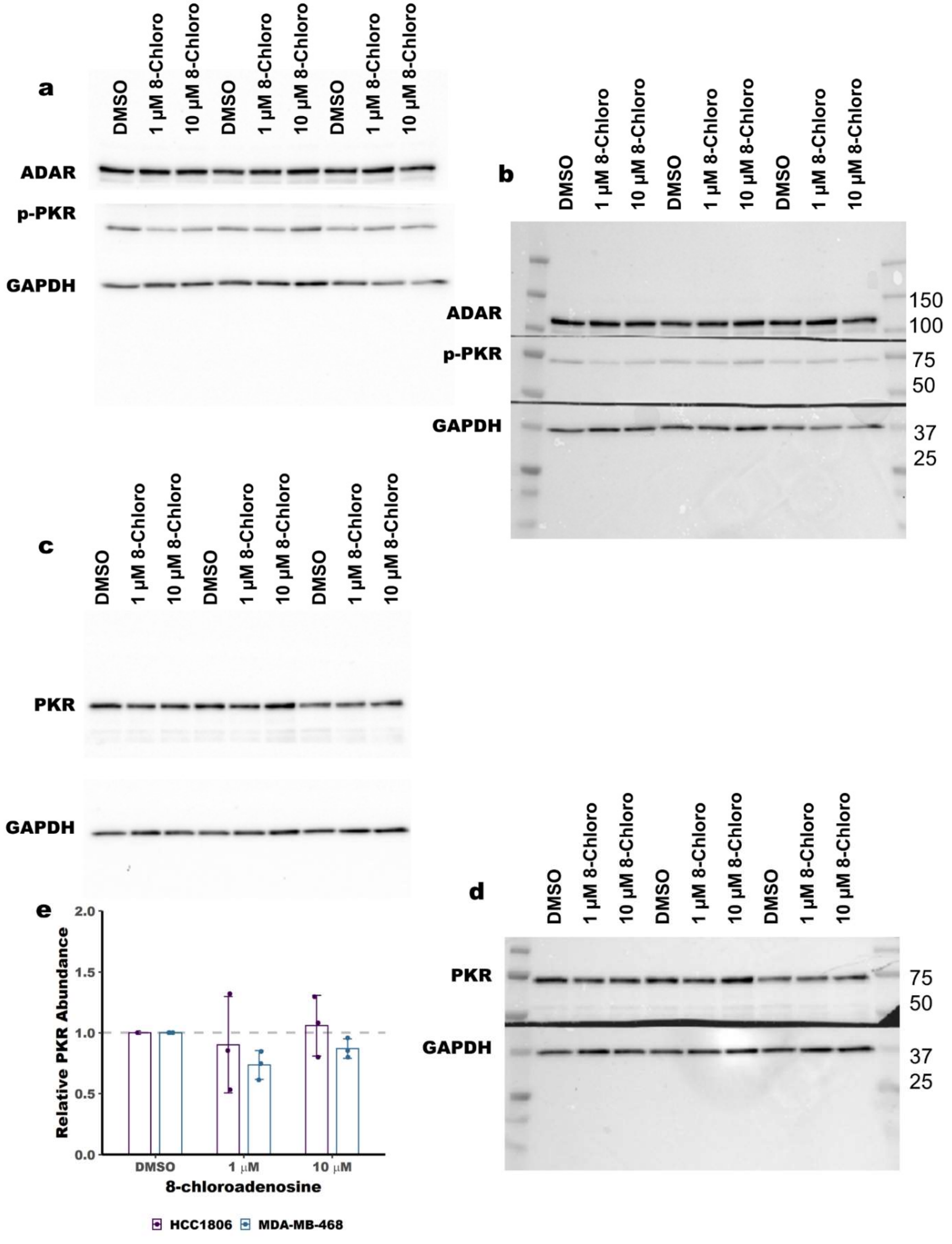

Supplemental Figure 2:

Uncropped immunoblots for MDA-MB-468 treatment with 8-chloroadenosine (8-chloro) associated with Figure 3b, 3e and 3f. Panels **a** and **b** are the uncropped chemiluminescence images, panels **b** and **d** are the chemiluminescence images merged with colorimetric images to show the molecular weight marker. **e** Quantification of PKR expression, PKR abundance was normalized to GAPDH and set relative DMSO.

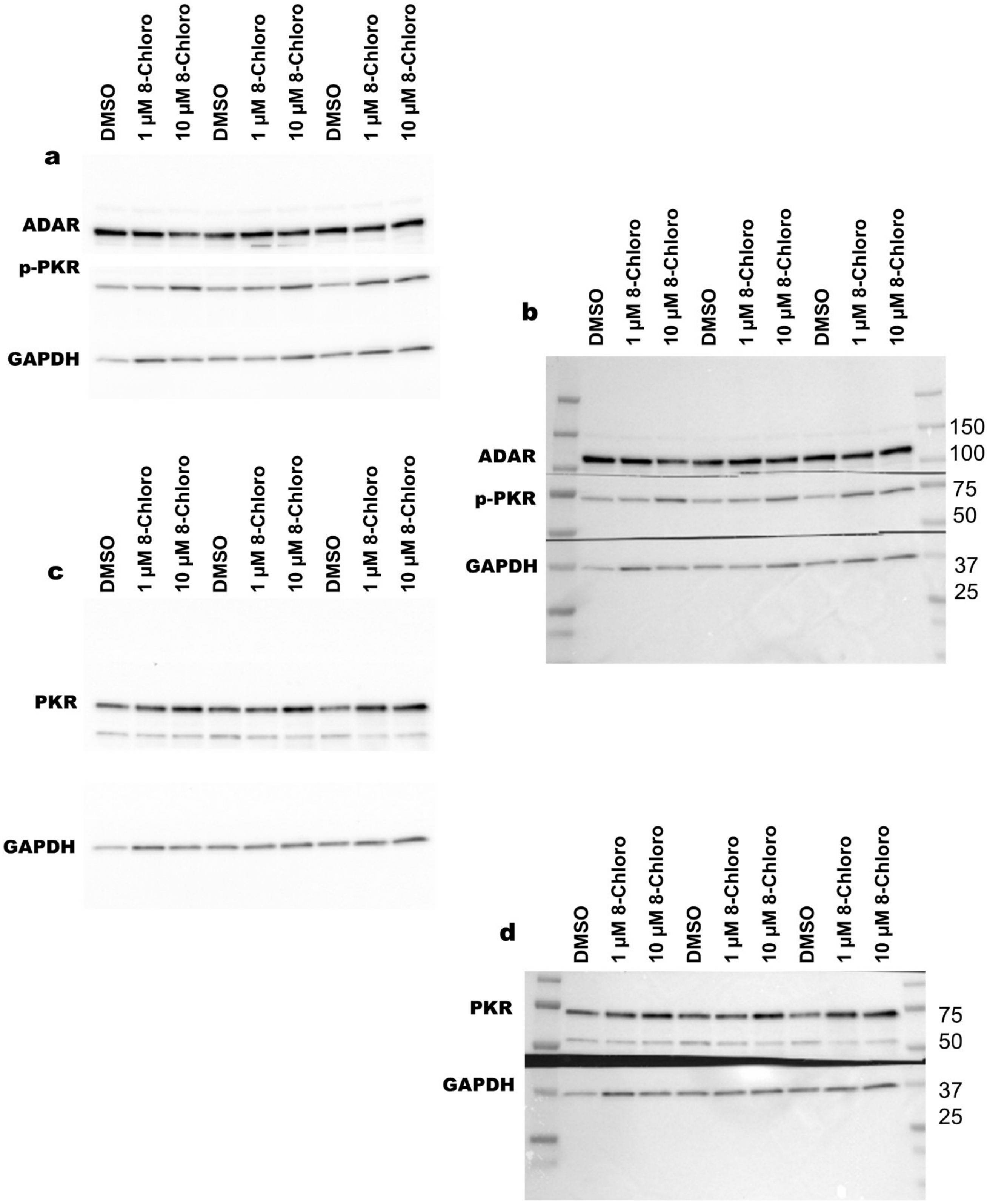

Supplemental Figure 3:

Uncropped immunoblots for HCC1806 treatment with 8-chloroadenosine (8-chloro) associated with Figure 3b, 3e and 3f. Panels **a** and **b** are the uncropped chemiluminescence images, panels **b** and **d** are the chemiluminescence images merged with colorimetric images to show the molecular weight marker.

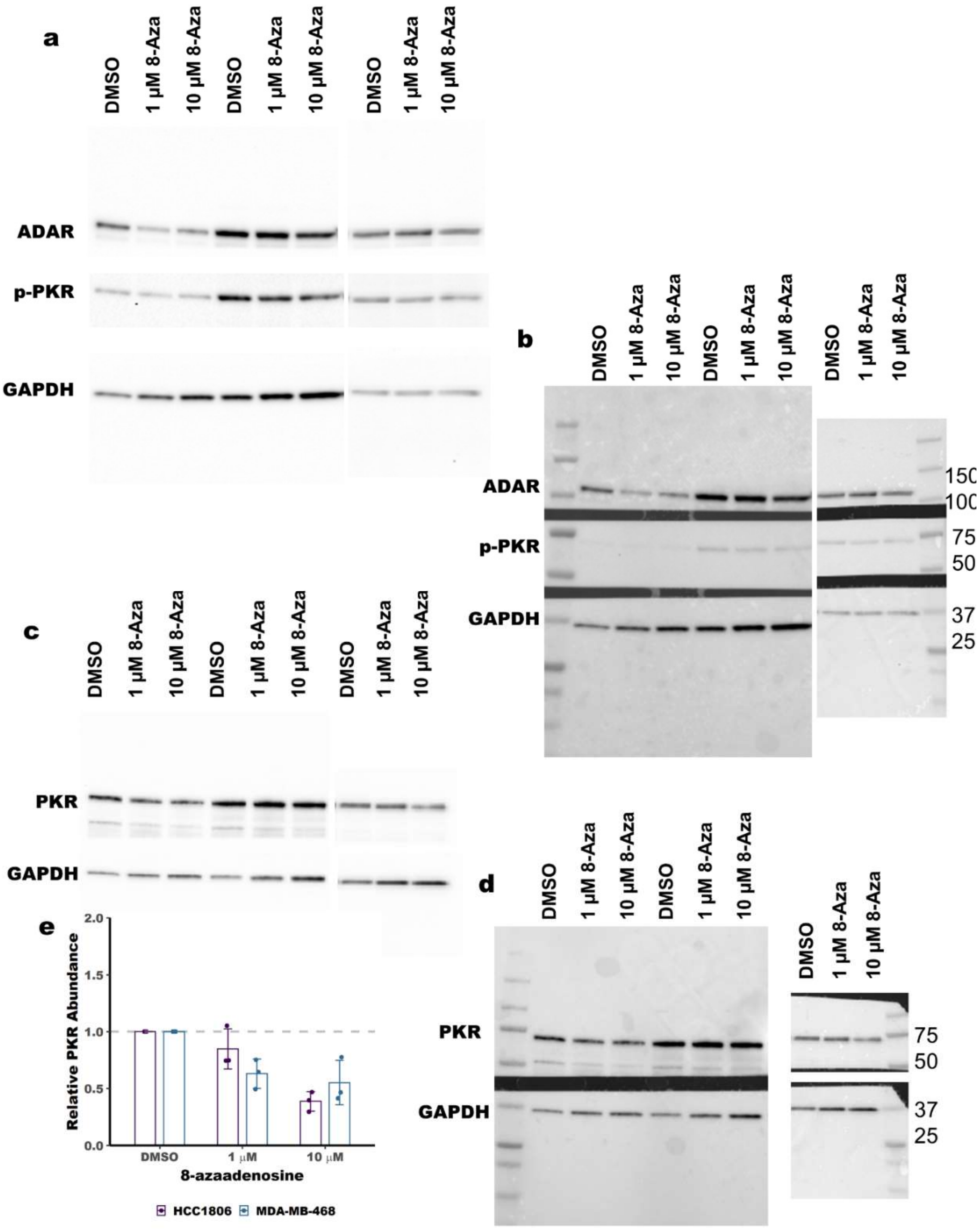

Supplemental Figure 4:

Uncropped immunoblots for MDA-MB-468 treatment with 8-azaadenosine (8-Aza) associated with Figure 3g-3i. Panels **a** and **b** are the uncropped chemiluminescence images, panels **b** and **d** are the chemiluminescence images merged with colorimetric images to show the molecular weight marker. **e** Quantification of PKR expression, PKR abundance was normalized to GAPDH and set relative DMSO.

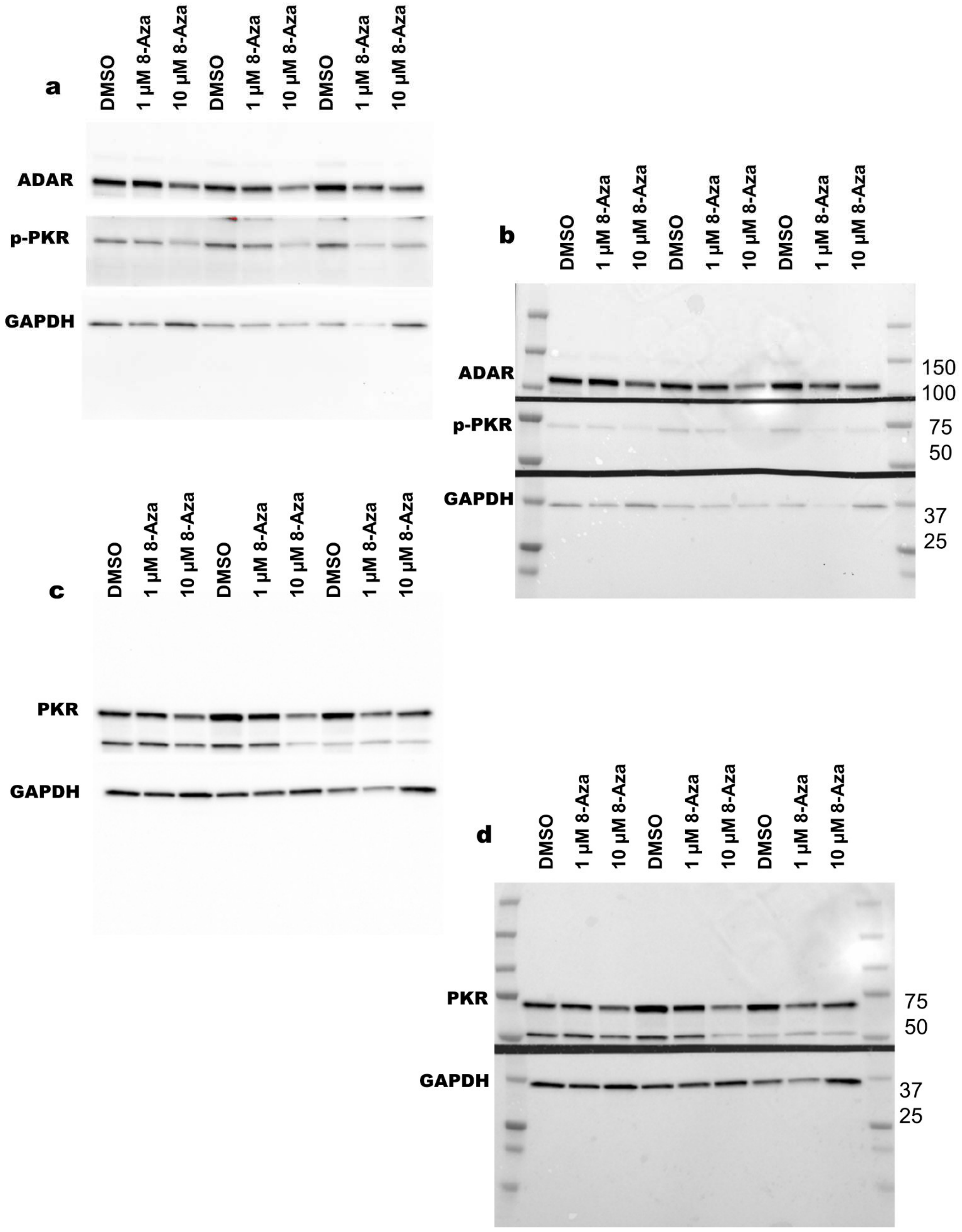

Supplemental Figure 5:

Uncropped immunoblots for HCC1806 treatment with 8-azaadenosine (8-Aza) associated with Figure 3g-3i. Panels **a** and **b** are the uncropped chemiluminescence images, panels **b** and **d** are the chemiluminescence images merged with colorimetric images to show the molecular weight marker.

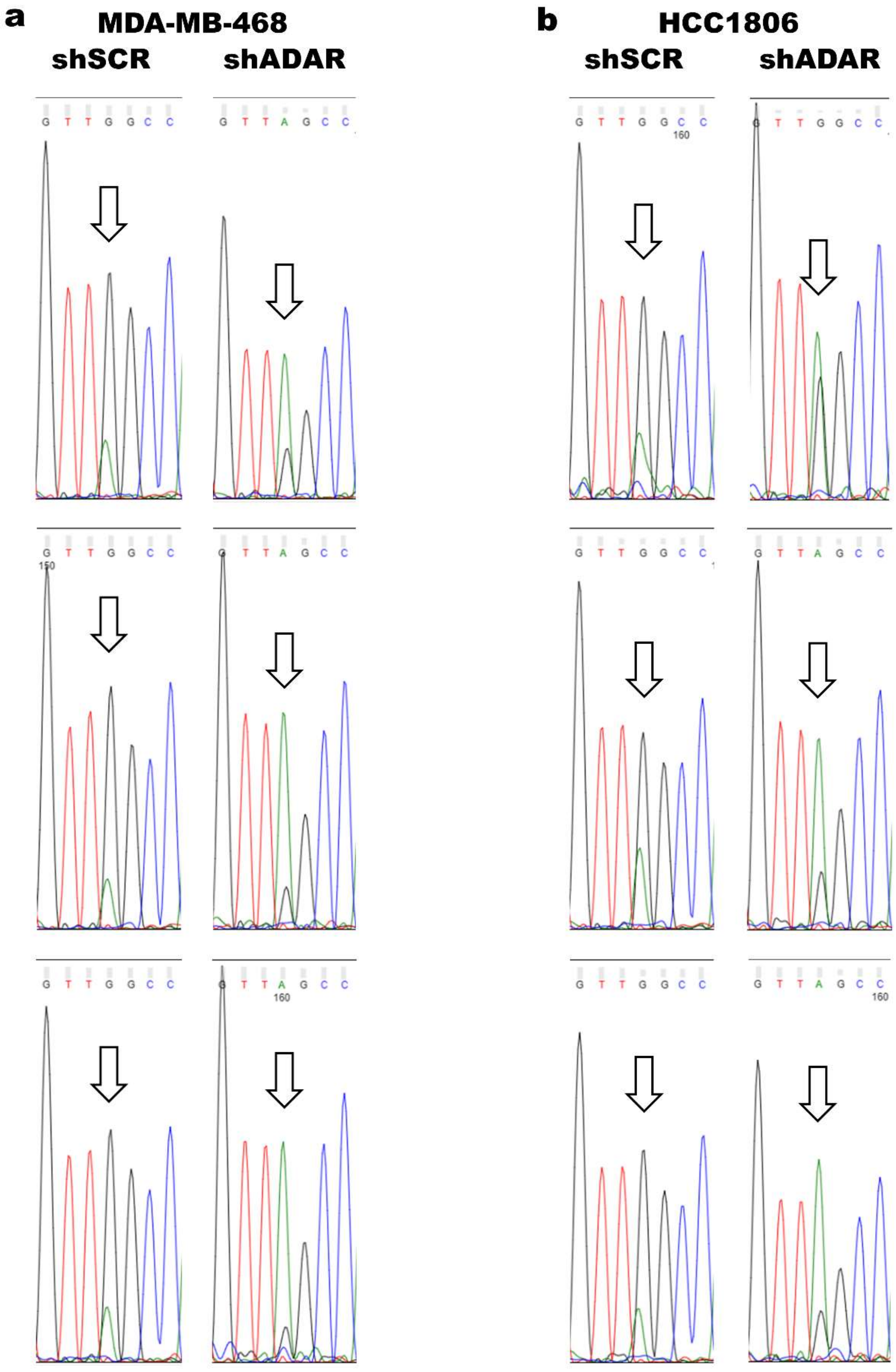

Supplemental Figure 6:

Chromatograms for all Sanger sequencing replicates associated with Figure 4a-b.

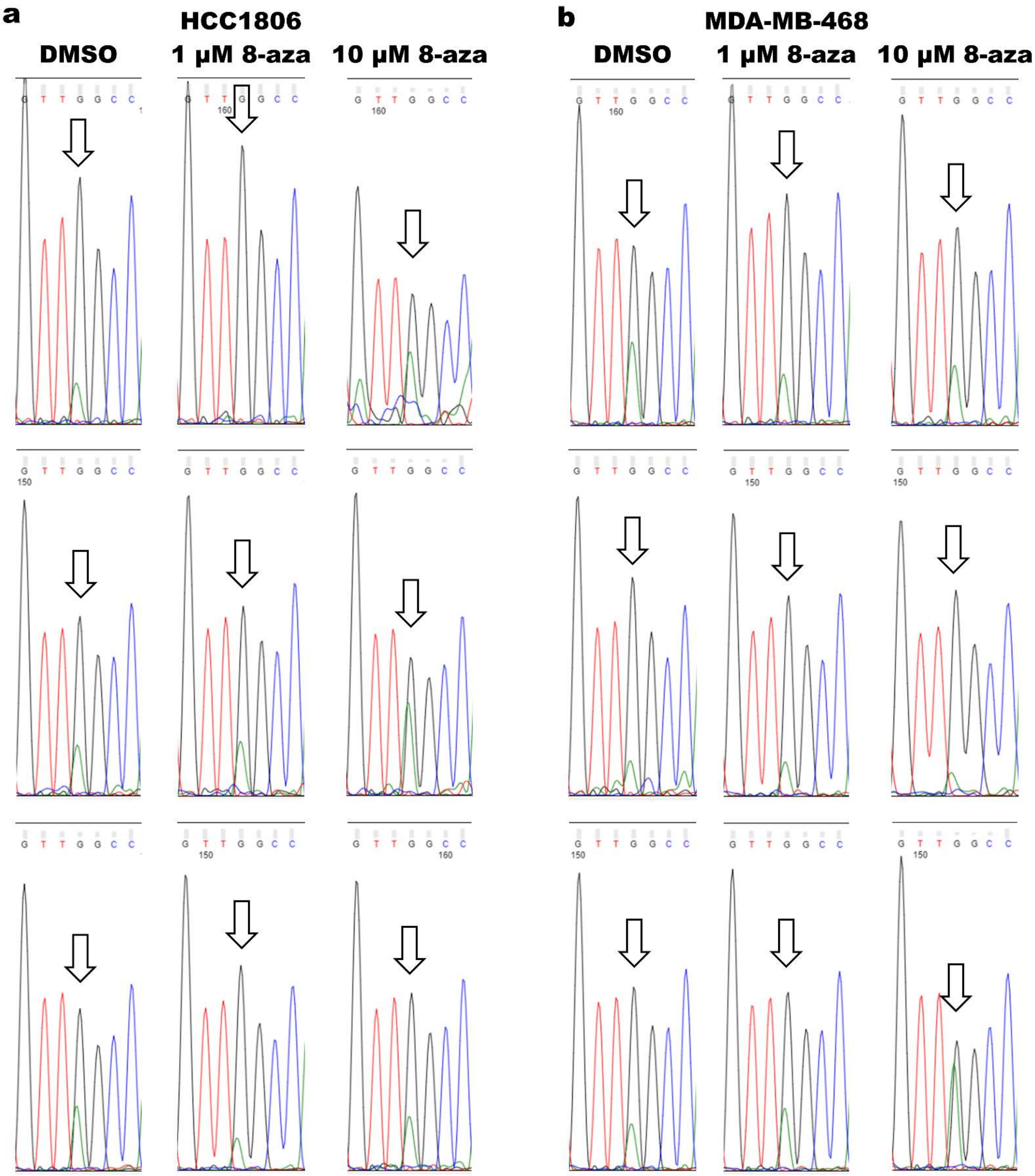

Supplemental Figure 7:

Chromatograms for all Sanger sequencing replicates associated with Figure 4c-d.

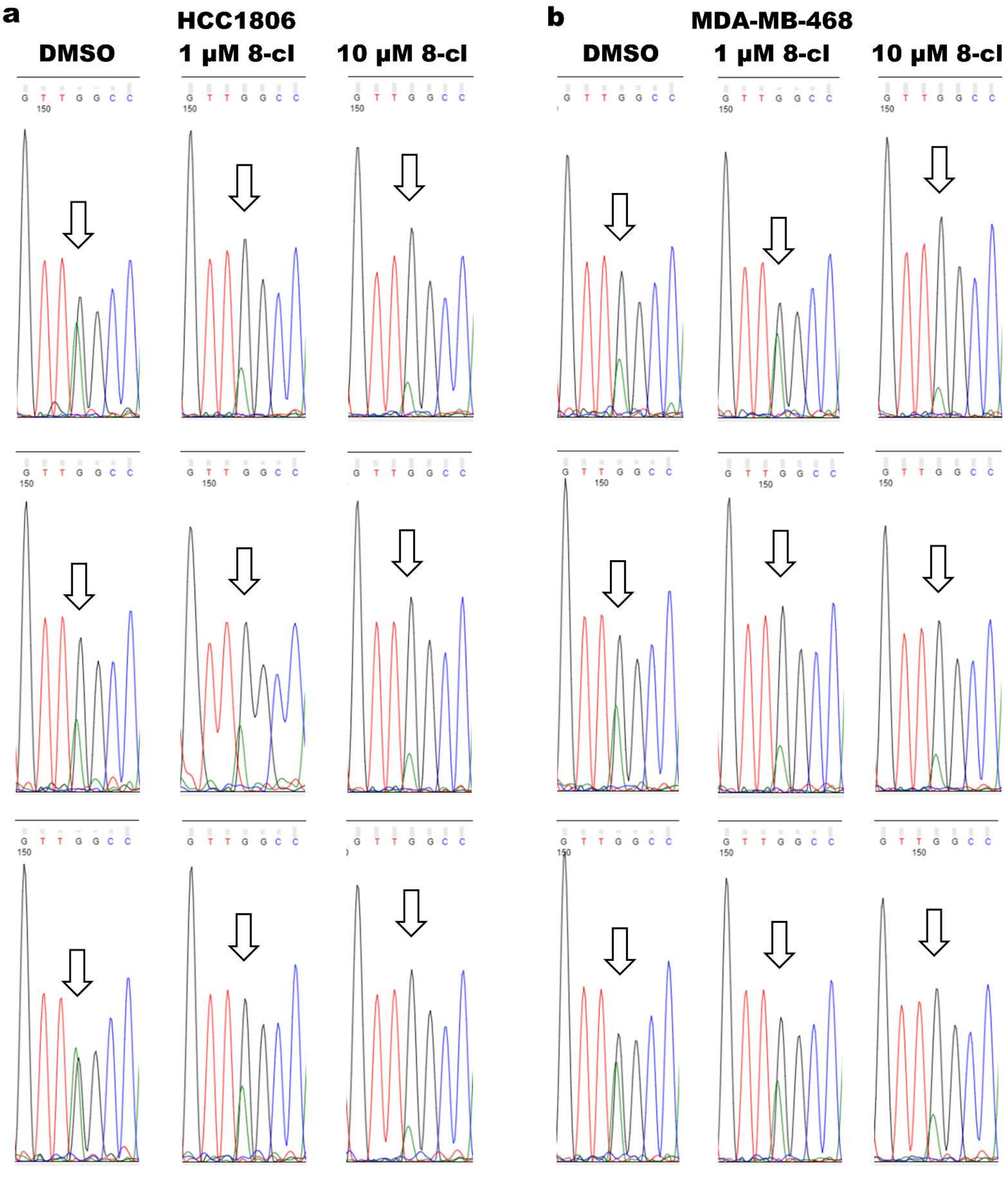

Supplemental Figure 8:

Chromatograms for all Sanger sequencing replicates associated with Figure 4e-f.

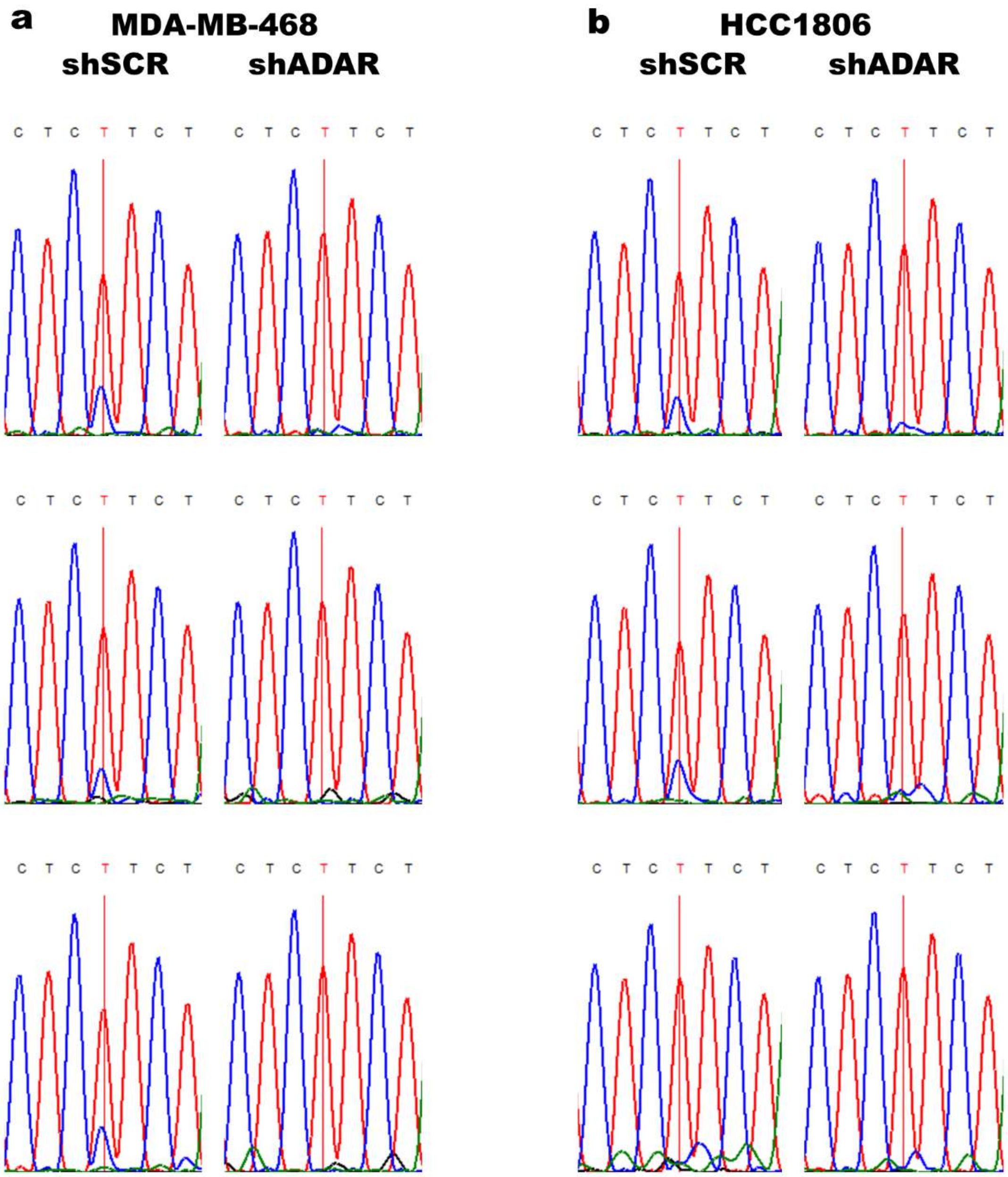

Supplemental Figure 9:

Chromatograms for all Sanger sequencing replicates associated with Figure 4g.

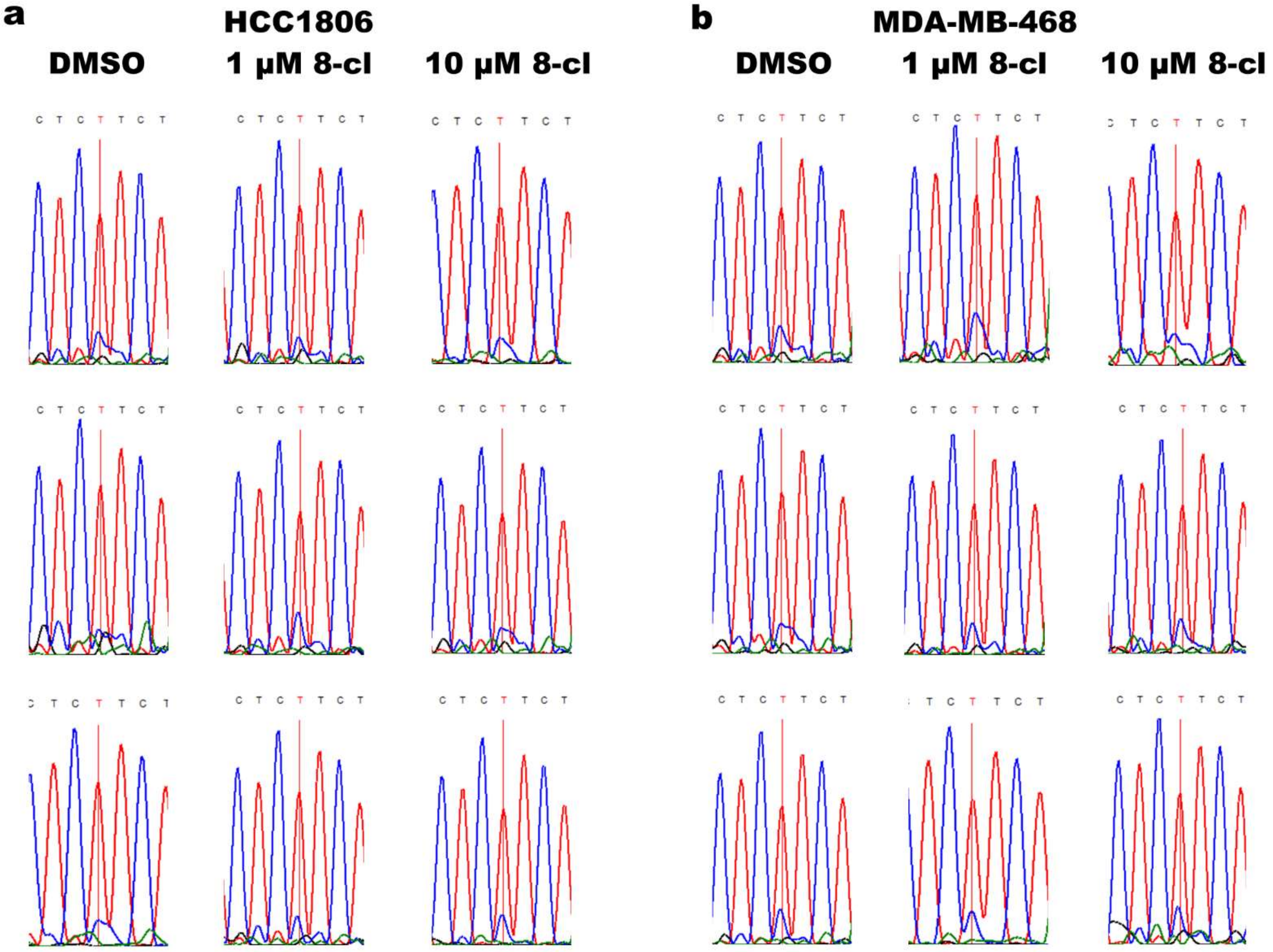

Supplemental Figure 10:

Chromatograms for all Sanger sequencing replicates associated with Figure 4h.

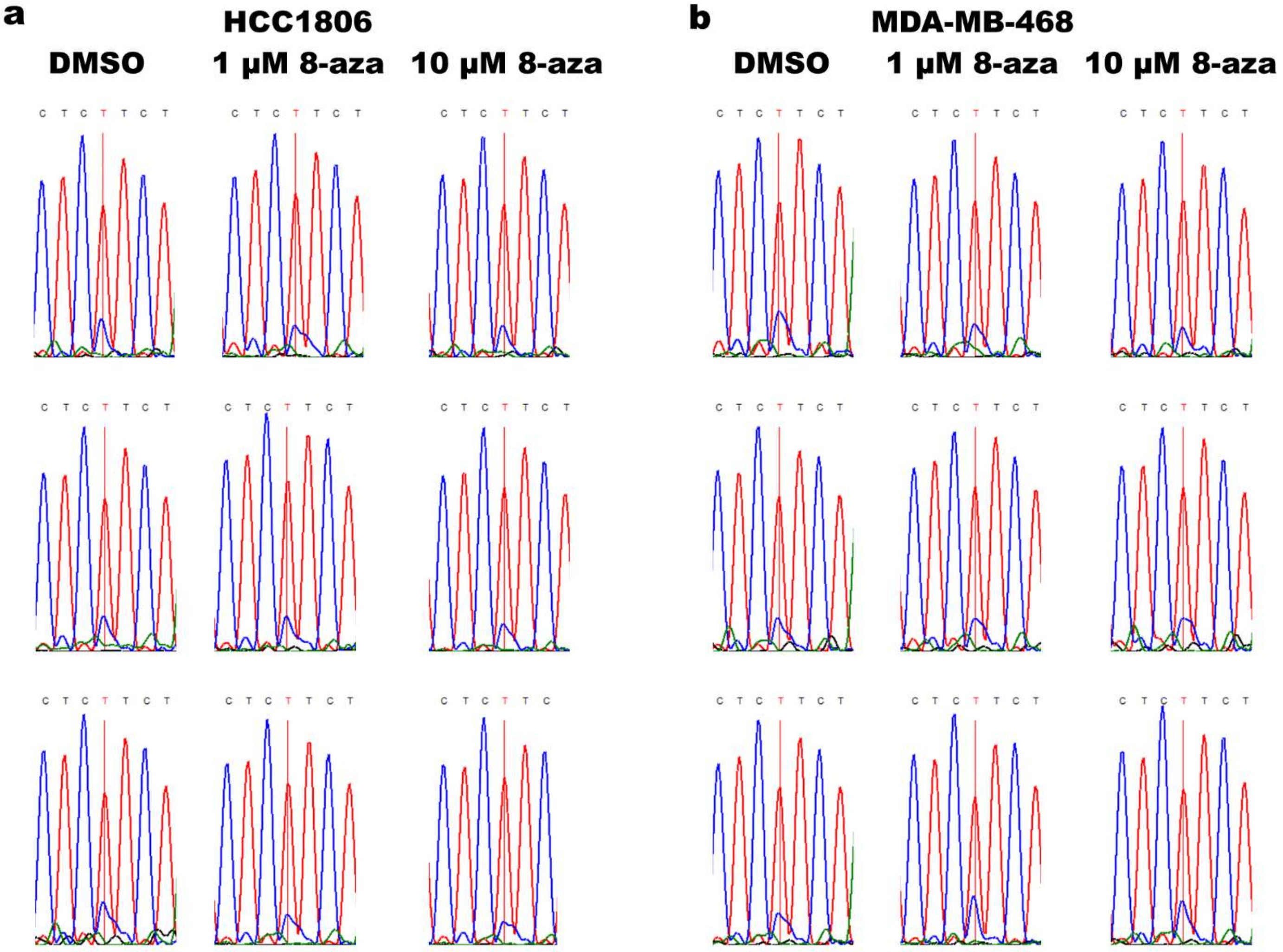

Supplemental Figure 11:

Chromatograms for all Sanger sequencing replicates associated with Figure 4i.

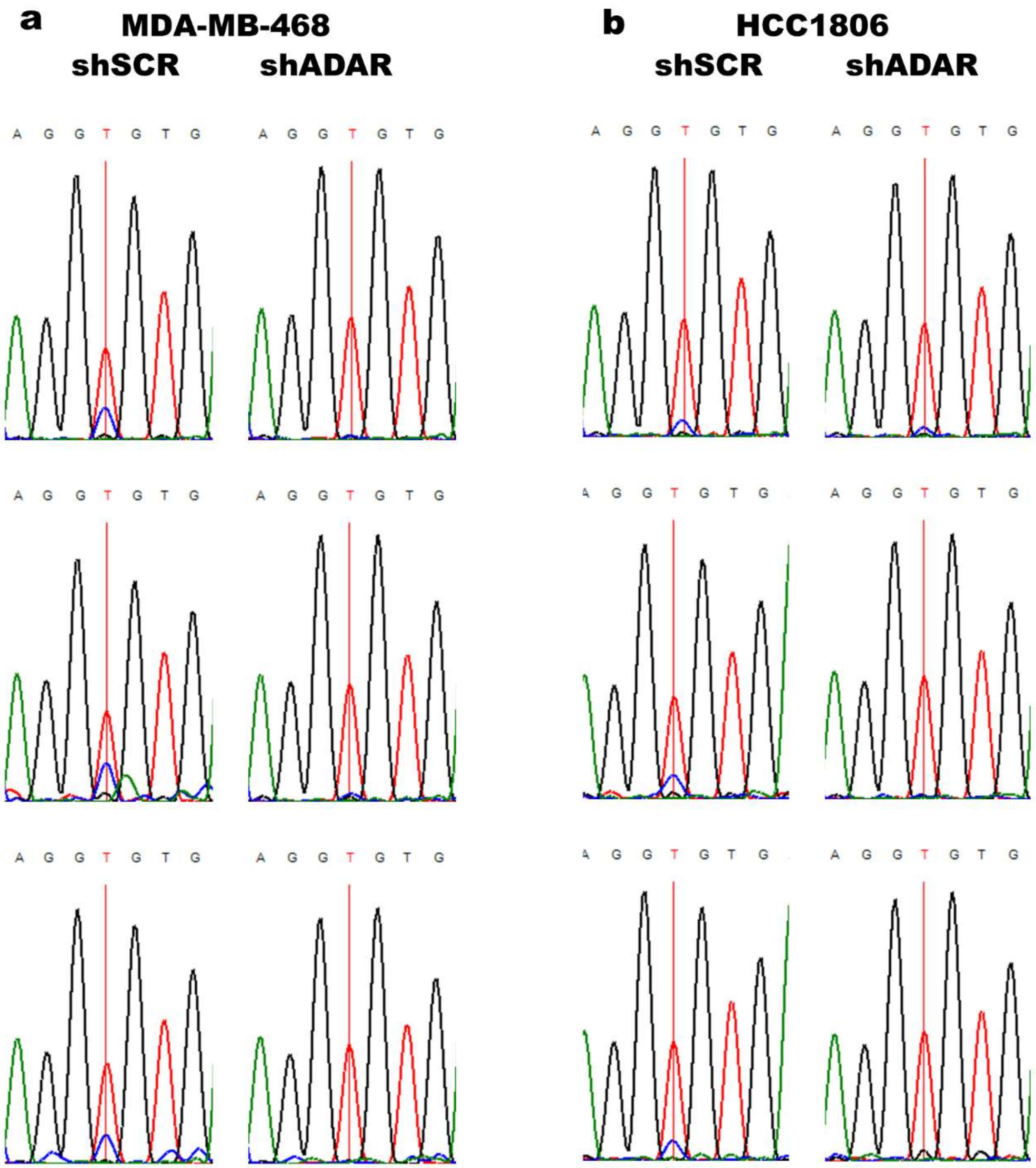

Supplemental Figure 12:

Chromatograms for all Sanger sequencing replicates associated with Figure 4j.

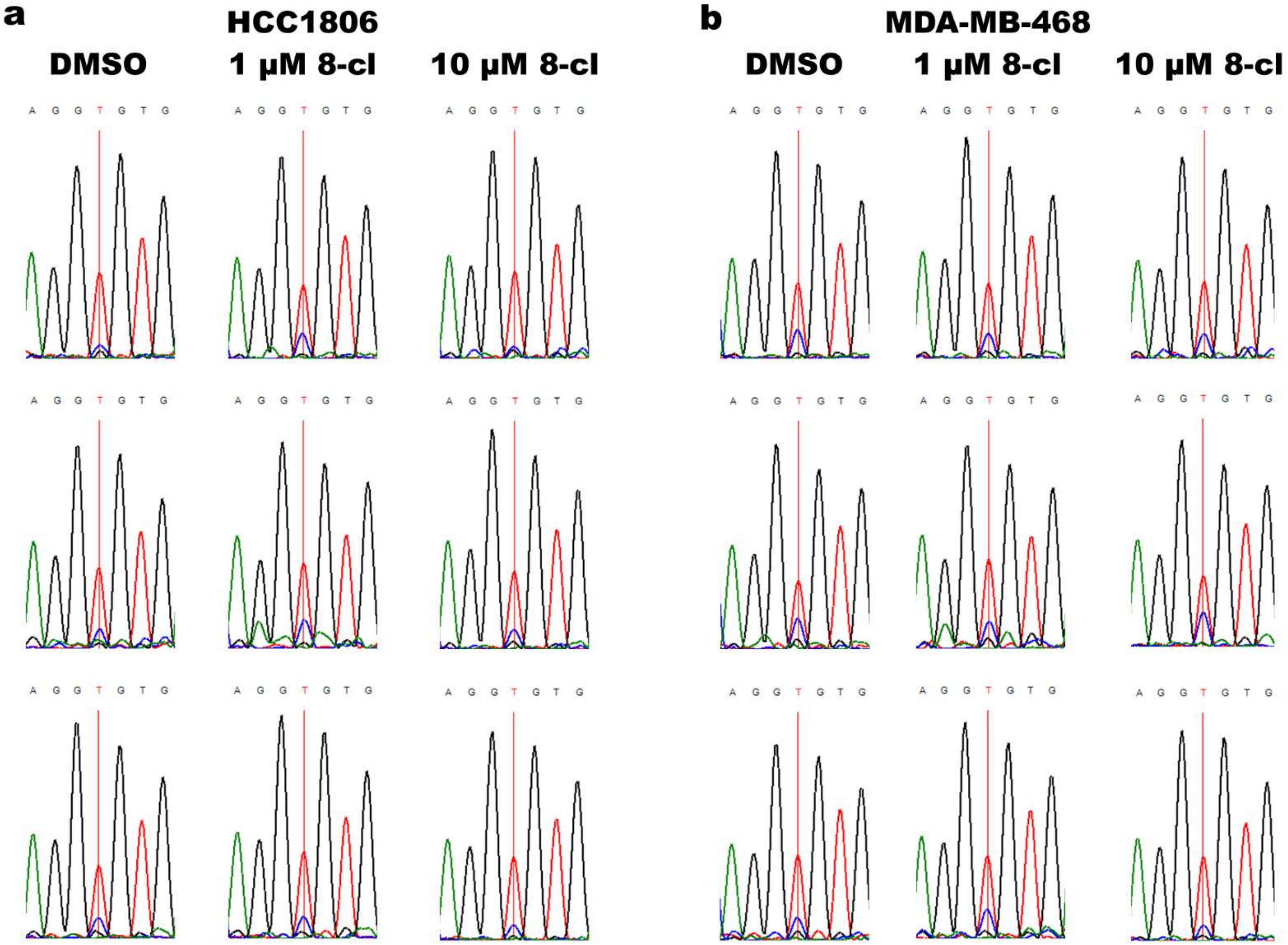

Supplemental Figure 13:

Chromatograms for all Sanger sequencing replicates associated with Figure 4k.

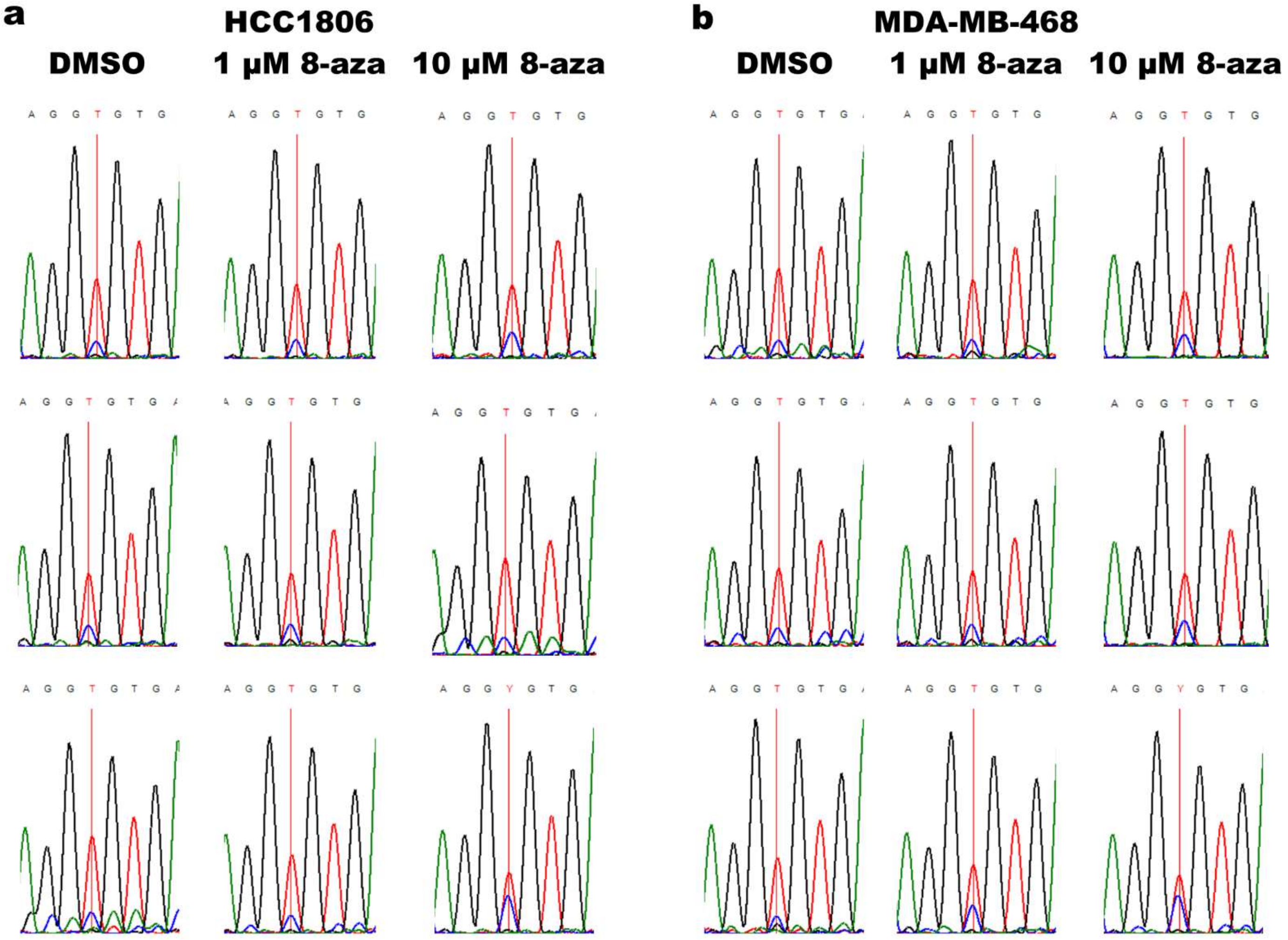

Supplemental Figure 14:

Chromatograms for all Sanger sequencing replicates associated with Figure 4l.

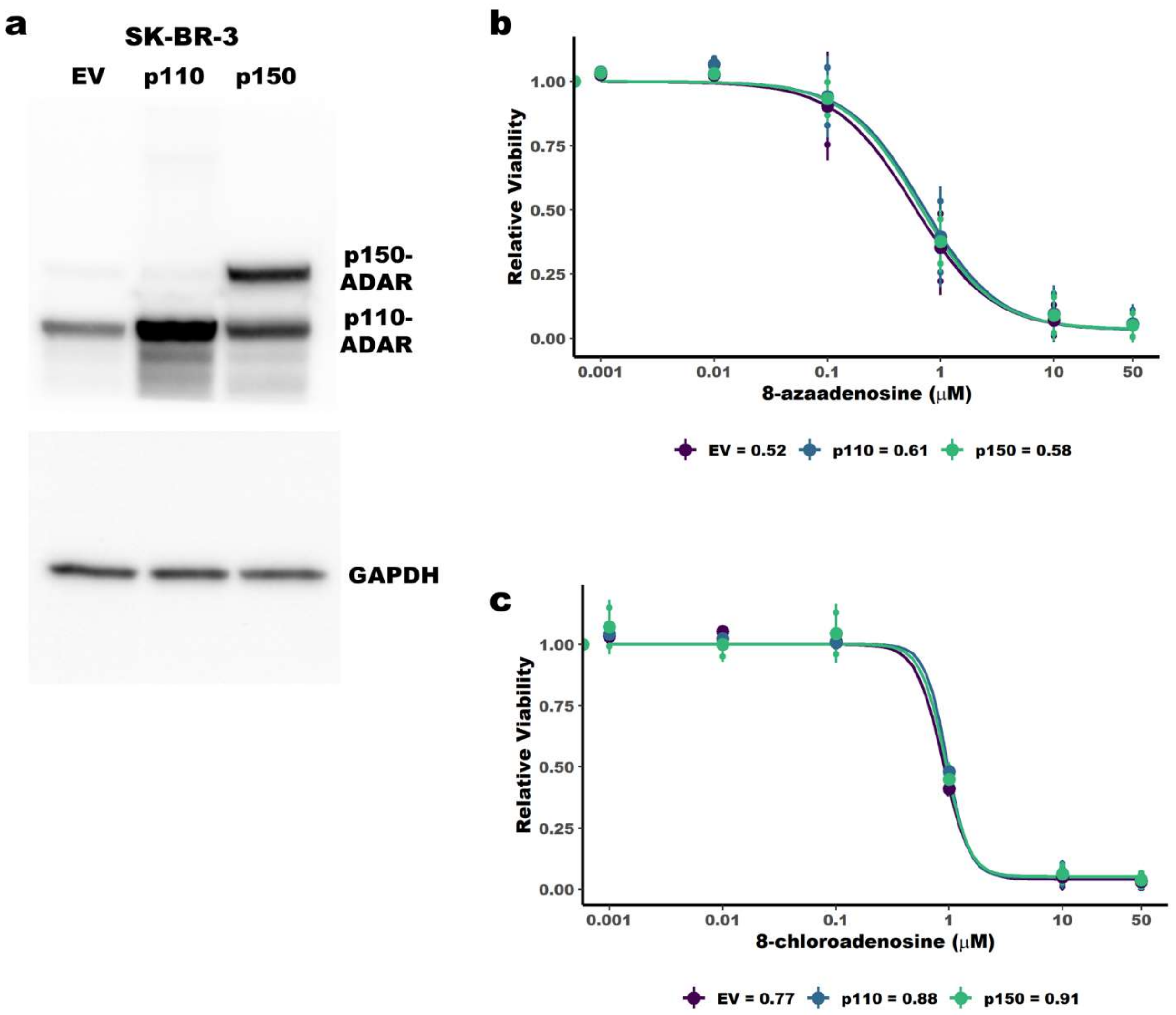

Supplemental Figure 15:

a Immunoblot showing overexpression of p110 and p150 ADAR in SK-BR-3. **b** and **c** Dose response curves for 8-azaadenosine and 8-chloroadenosine in SK-BR-3 cells with (p110 or p150) or without (EV) overexpression of ADAR. In panels **b**, and **c** the large points are the mean of two independent experiments, the smaller points are the mean of three technical replicates performed for each experiment, error bars are mean +/- standard deviation.
